# Functional Clustering of Metabolically Related Genes Is Conserved across *Dikarya*

**DOI:** 10.3390/jof9050523

**Published:** 2023-04-28

**Authors:** Gina M. Cittadino, Johnathan Andrews, Harpreet Purewal, Pedro Estanislao Acuña Avila, James T. Arnone

**Affiliations:** 1 Department of Biological and Environmental Sciences, Le Moyne College, Syracuse, NY 13214, USA; 2 Universidad Tecnológica de Zinacantepec, San Bartolo el Llano, Zinacantepec 51361, Mexico

**Keywords:** secondary metabolites, biosynthetic gene clusters, transcriptional co-regulation, genome organization, position effects, *Dikarya*, *Basidiomycetes*

## Abstract

Transcriptional regulation is vital for organismal survival, with many layers and mechanisms collaborating to balance gene expression. One layer of this regulation is genome organization, specifically the clustering of functionally related, co-expressed genes along the chromosomes. Spatial organization allows for position effects to stabilize RNA expression and balance transcription, which can be advantageous for a number of reasons, including reductions in stochastic influences between the gene products. The organization of co-regulated gene families into functional clusters occurs extensively in *Ascomycota* fungi. However, this is less characterized within the related *Basidiomycota* fungi despite the many uses and applications for the species within this clade. This review will provide insight into the prevalence, purpose, and significance of the clustering of functionally related genes across *Dikarya*, including foundational studies from *Ascomycetes* and the current state of our understanding throughout representative *Basidiomycete* species.

## 1. Background and Introduction

Approximately one billion years ago, the fungal lineages emerged, ultimately evolving into a large, diverse kingdom of eukaryotic organisms containing species commonly referred to as yeasts, molds, smuts, rusts, lichens, and mushrooms [1]. Although this kingdom is composed of heterotrophic organisms that span a wide range of habitats, fungi are diverse organisms that cannot be easily characterized [2]. Representative members include the budding yeast, *Saccharomyces cerevisiae* (an extensively studied model organism commonly known as baker’s and brewer’s yeast), numerous pathogens, and delectable mushrooms. The lifestyles, habitats, and niches occupied by fungi are incredibly diverse, including species that can be both unicellular and multicellular, with the ability to reproduce either sexually or asexually (or both) [3,4]. Fungi constitute an invaluable place within the ecosphere, where they serve many natural roles and act as decomposers, symbionts, and pathogens [5,6].

Extensive efforts have been taken to identify and characterize the membership of this kingdom using diverse methodologies (technological advances have been integral to expanding these efforts). Despite this, only a fraction of the predicted members of the fungal kingdom have been identified, and even fewer have been characterized. Scanning the literature over the past decades, the predicted number of fungal species proposed to exist has gone up by several orders of magnitude—predictions were in the range of around 100,000 in the 1940s, they increased to 250,000 in the 1950s, they expanded to over 700,000 in the 2000s, to 1.5 million in the early 1990s, and today’s predictions place the number in the range of approximately 5.1 million species [7,8,9,10,11]. Different approaches at modeling and estimating the numbers result in drastically different estimates. One rigorous study places the number of species of fungi in a conservative range of 2.2 to 3.8 million species [12]. On the less conservative end of the spectrum, a recent study has predicted that there are upwards of one trillion microbial species and implies that the actual number of fungal species could be significantly higher, potentially by several orders of magnitude [13]. Regardless of whatever the true number may be, only a fraction of the fungal kingdom has been identified and characterized [14]. Those that have been studied thus far have revealed a wealth of information and a staggering amount of diversity, along with myriad applications [15].

Fungi have a diverse and complex metabolism, as well as specializations that have enabled them to adapt to an incredible range of habitats throughout the planet. Fungi have the ability to survive (and thrive) in marine environments, alpine tundras, the deep ocean, and Antarctic soils, to name just a few of the more extreme environments where fungi are found [16,17,18,19,20]. These adaptations have driven members of this kingdom to evolve a diverse repertoire of bioactive molecules. Hundreds of fungi have been studied as a source of compounds for pharmaceutical, agricultural, and industrial uses [21]. At present, it is estimated that up to half of the entire commercial repertoire of enzymes are derived from fungal origins [22]. Fungi are a source of bioactive molecules and compounds that are antibacterial antibiotics, antimycotics, antiviral, and anti-cancer agents [23]. Other members of this clade enhance agriculture and crop production through their demonstrated abilities to control plant diseases and pests, including insects, nematodes, weeds, and post-harvest diseases [23]. As studies have expanded and extended, fungal products are being applied in new, innovative ways. These applications are taking place in emerging fields, such as architecture, where fungi can be used as an inspiration for design and as a component in building materials [24]. In light of the fact that, to date, only a fraction of the fungal diversity predicted to exist has been characterized, further efforts to expand the identification and characterization of fungi represent potential sources of innumerable future developments and applications.

There is as much variability within the fungal kingdom as there is found across the plant and animal kingdoms [25]. Fungi are subdivided into five distinct phyla: the *Ascomycetes*, *Basidiomycetes*, *Chytridiomycetes*, *Glomeromycetes*, and *Zygomycetes*. *Ascomycetes* and *Basidiomycetes* are paired together as the subkingdom *Dikarya* because these fungi have dikaryotic hyphae, the filamentous network that multicellular fungi can form [26]. Additionally, known as ‘higher fungi,’ these two closely related phyla are the source of many useful bioactive compounds. Members of this clade can be easily genetically manipulated, offering opportunities for their employment as ‘cell factories’ for the enhanced production and biosynthesis of molecules with many medical and pharmaceutical uses [27,28].

**Figure 1:**
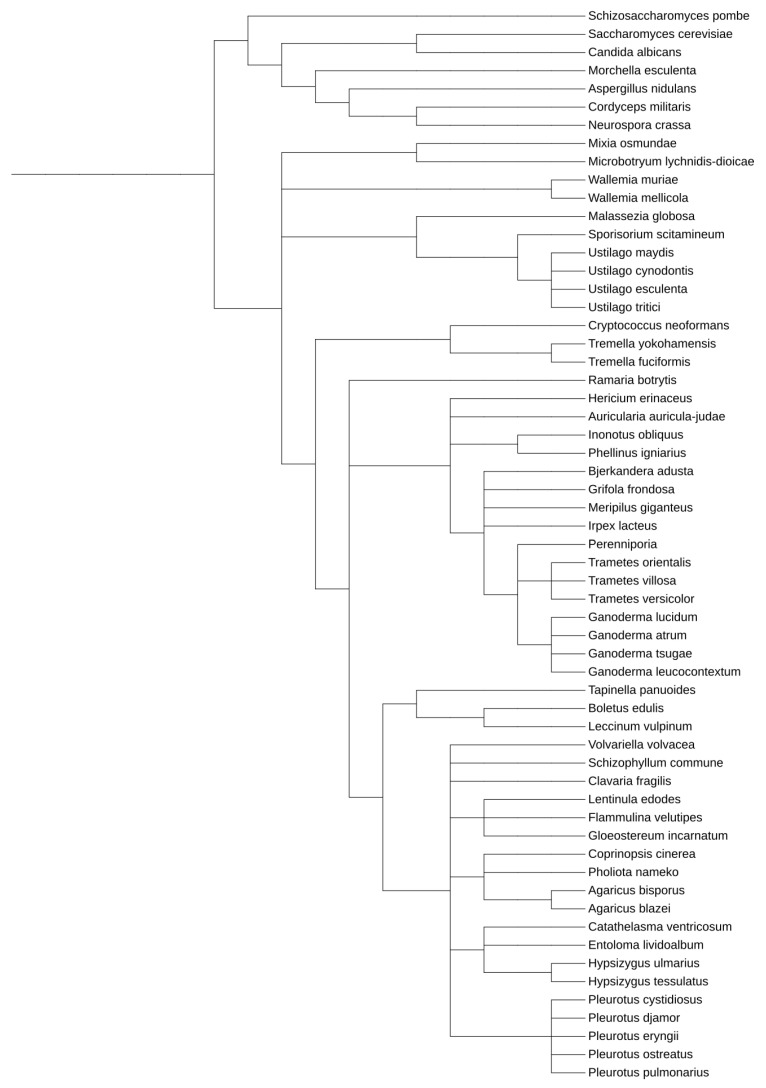
Phylogenetic relationship between model *Ascomycetes* and the *Basidiomycetes* discussed within this work. This tree was generated utilizing the Interactive Tree of Life tool, using NCBI taxonomy inputs [34]. A whole genome duplication event occurred after *S. cerevisiae* and *C. albicans* diverged.

Within *Dikarya*, the *Ascomycetes* are much better understood and characterized than their closely related *Basidiomycetes* brethren (Figure 1 and Supplementary Figures S1 and S2), although the latter contains many species of broad interest for pharmaceutical, biotechnological, medical, and agricultural applications and study (Supplementary Table S1). Many *Ascomycetes* are widely utilized as model systems for myriad eukaryotic molecular and genetic processes, including *Saccharomyces*, *Aspergillus*, *Neurospora*, *Schizosaccharomyces*, and *Candida* species [29,30,31]. The first eukaryotic organism to have its genomes sequenced was an *Ascomycete*, and currently, there are well over 1000 species within this clade that have been sequenced, allowing for exceptional comparative genomics study and analysis [32,33].

This has led to a comprehensive understanding of the extensive role functional clustering plays in shaping the organization and expression of gene families [35]. The focus of this review is to provide insights into the prevalence, purpose, and significance of the clustering of functionally related genes across *Dikarya* (including foundational studies from *Ascomycetes*) and to describe the current state of our understanding throughout representative *Basidiomycete* species. We will not address rDNA tandem repeats or the mating-type loci clusters and groupings, as those have been extensively characterized elsewhere due to their exceptionally high incidence of conservation [36,37,38]. Although these loci are widely conserved, they fall outside of the scope of this review.

## 2. Within *Dikarya*, Study of *Ascomycetes* Has Yielded Complex Insight into the Roles of Spatial Positioning on Gene Expression and Genome Organization

*Ascomycetes* include several model organisms that have long been used for the study of myriad molecular processes, including the S. *cerevisiae*, the opportunistic pathogen *Candida albicans*, and the fission yeast *Schizosaccharomyces pombe* [31,39,40]. Their widespread adoption and use has led to the availability of high-quality genomes for analysis much earlier than for other model systems [41]. This prompted the early use of transcriptomics—powerful gene expression studies conducted using microarray and RNA-sequencing technologies across many environmental and stress responses [42,43,44]. An interesting observation began to emerge from the early application of transcriptome analysis, as the genome appeared to be organized into domains of correlated gene expression [45].

The *GAL* gene cluster was one of the first co-expressed metabolic gene clusters to be identified in *S. cerevisiae* [46]. The three genes that comprise this cluster, *GAL7-GAL10-GAL1*, are found grouped together as a triplet on Chromosome II, along with multiple cis-regulatory DNA sequences necessary for their transcriptional regulation [47,48]. These genes are coordinately regulated to allow the cell to modulate expression of the entire cluster for the metabolism of lactose in a fast and efficient way. This regulation involves multiple steps, including trans-acting DNA binding proteins and chromatin remodeling enzymes [49,50]. Linkage of these genes is vital to organismal survival, as the galactose metabolic pathway involves the transient production of a toxic metabolite, galactose-1-phosphate (Gal-1-P), by the enzyme Gal1p. This toxic metabolite is converted to glucose-1-phosphate by Gal7p. This is essential, as the deletion of the *GAL7* gene exhibits much slower growth and reduced levels of fitness [51]. The clustering of these genes is thought to assist in buffering stochastic influences on their expression, minimizing the accumulation and cytotoxicity associated with Gal-1-P. Uncoupling the expression of these genes from endogenous loci leads to increased toxin accumulation, cytotoxicity, and cell death [52]. Minimizing toxic intermediary molecules can exert a selective pressure that biases genome organization for some metabolically related gene families into clusters. Such an arrangement would effectively buffer clusters from stochastic effects upon gene expression, preventing the build-up of toxic intermediates.

The early observation/characterization that the galactose metabolic genes were co-localized along the chromosome was built upon, leading to the observation that the budding yeast’s genome contained many functionally related gene clusters. The ribosomal rRNA and ribosome biosynthesis (RRB) genes were one of the first transcriptionally co-expressed gene families found with a significant fraction of the composite members clustered throughout the genome [53]. Subsequent study revealed that this arrangement is a feature of the ribosomal protein (RP) gene family as well. These clusters are primarily found as pairings and occasionally triplets, and this organization into clusters is a defining feature of both regulons. Additionally, this distribution is conserved from *S. cerevisiae* to both *C. albicans* and *S. pombe*, and even outside of the fungal kingdom, to a wide variety of eukaryotic organisms [54,55].

As the RRB and RP regulons were some of the earliest gene families that were identified to exhibit a non-random genomic distribution, this significance has been functionally dissected at the molecular level. The RRB gene pair, *MPP10-MRX12*, are clustered together on chromosome X and share promoter elements that are present upstream of *MPP10* only [53]. Targeted mutagenesis to effectively eliminate these promoter motifs disrupted the transcription of both genes from the rest of the RRB gene family. Separating this gene pair via the insertion of a gene (a leucine biosynthetic coding sequence) was effectively able to uncouple the regulation of *MPP10* from *MRX12* [56]. The RRB genes are special in that they are required in roughly stochiometric levels to help coordinate the production of ribosomes, which are major consumers of intracellular resources [57]. It is possible that the pairings of genes that participate in a shared metabolic pathway, such as ribosome biogenesis, allows for tighter transcriptional regulation, minimizing wasteful energetic expenditure in the fluctuations of individual components. This may result in selective pressure favoring the formation of clusters. This is simultaneously quite distinct from and reminiscent of the organization of related metabolic families into operons—a phenomenon that is observed extensively in prokaryotes [58].

Systematic analysis of the incidence of functional clustering of metabolically related groupings defined by gene ontology designations found that the grouping of members into pairings was a feature of many gene families in *S. cerevisiae* (27% of all families exhibited a statistically significant incidence of clusters) [59]. An extension of this analysis to the more distantly related *C. albicans* revealed the same results, in spite of the evolutionary distance—and drastically different lifestyles—between these two species [60]. One surprising discovery was that, although a similar phenomenon was observed, the actual members that comprised the pairings were different between the species. This indicates that the functional grouping of genes throughout the genome is not simply the result of an ancestral relationship that has been subsequently maintained throughout evolution. This suggests that the formation of clusters may be random in nature (e.g., the result of gene duplications, recombination events, etc.), but once formed, there is a selective advantage to maintaining the pairing.

The lineage that gave rise to *S. cerevisiae* underwent a whole genome duplication (WGD) event—a rare phenomenon whereby the entire genetic content of an ancestral cell was effectively doubled sometime after the split from the *Kluyveromyces* lineage, which took place approximately 150 million years ago [61,62]. This WGD was followed by the loss of most of the duplicates and evolutionary divergence between many of those that were retained. The number of protein-coding open reading frames retained revealed that 13% of the duplicated genes from this event had been maintained, while the rest were lost [61]. An analysis of the effects of the WGD on the prevalence of functionally clustered metabolic genes within the ribosomal protein and RRB genes in *S. cerevisiae* determined that this genetic event had a negligible effect on the formation of functional clusters within these gene families [55]. The RP genes are present in multiple copies and are frequently clustered together throughout the *S. cerevisiae* genome [54]. The only effect of the WGD event on the RP and RRB gene families was the duplication and maintenance of a RP gene pairing, whose clustering predated this event (*RPL18A-RPS19A* and *RPL18B-RPS19B*), and they have been separately maintained as dual clusters throughout the intervening time since [55]. Thus, this evolutionary event did not appear to be influential in the formation of these functional clusters.

*S. cerevisiae* revealed many surprising findings regarding the interconnected nature of transcription throughout chromosomal regions close in spatial proximity. The yeast knockout collection allowed for rapid characterization of the phenotypes associated with loss-of-function mutations across the genome [63]. This collection was a systematic effort that resulted in the disruption of non-essential genes throughout the genome a via PCR-mediated homologous recombination that inserted a kanamycin resistance (*KAN^R^*) marker, as well as associated regulatory elements to drive transcription, effectively deleting each gene sequentially. Systematic analysis found that between 7–15% of these annotations were attributed incorrectly due to transcriptional interference by the *KAN^R^* reporter gene chromosomal region surrounding the locus of integration [64]. The integration of the *KAN^R^* marker disrupted the expression of the neighboring gene, supporting a model whereby local spatial positioning and gene order directly affect transcription throughout a genomic region, potentially playing a regulatory role in the coordination of transcription at a specific locus. This phenomenon is commonly referred to as the ‘neighboring gene effect’ (NGE) and ultimately appeared to contaminate about 10% of all attributed phenotypes across analyzed datasets, requiring re-annotation and analysis [65].

The NGE is essentially an extension of the role that chromosomal position effect plays on gene expression, and this phenomenon that has been well-documented among genetic researchers. Chromosomal position effects result in the silencing of integrated reporter genes proximal to heterochromatic regions in many organisms and are referred to by the names ‘telomere proximal effect’ and ‘position effect variegation’ [66,67]. This effect has been found to be a characteristic that is conserved throughout eukaryotes, including humans [68]. One consequence of these effects is that they may provide a potential evolutionary mechanism that underlies the formation and maintenance of metabolic clusters. Whenever a functionally related pairing forms, the co-expression of a cluster may be selected for by evolutionary mechanisms favoring this arrangement, and this is a consequence of the position effects that occur upon each other.

The ease of genetic manipulation of the budding yeast has subsequently led to a number of systematic genetic libraries, including efforts to tag every open reading frame with a green fluorescent protein and TAP (tandem affinity purification) tags to large-scale insertions of marker and reporter genes [69,70]. Such resources allow for continued systematic analyses and offer insight into the role of position effects on transcription throughout the genome. One large scale analysis characterized the role of position effects on the expression of a GFP reporter throughout the genome at approximately 500 loci, finding that the integration site led to a twenty-fold difference in the levels of expression [71]. This was verified and expanded independently using a red fluorescent protein (RFP) construct integrated at over 1000 different loci. This analysis measured a thirteen-fold difference in expression due to position effects exerted on the site of integration [72].

Gene expression analyses that eschew the use of reporters have painted a compelling picture of the impacts that position effects can have and have helped to establish a hypothesis that provides insight into the location of functional clusters for gene expression. There is a weak, global correlation that exists across the genome in *S. cerevisiae*—the closer that any two genes are along a chromosome, the higher the correlation between their expression [73]. There is significant variability across the genome, as some regions exhibit incredibly tight, correlated co-expression patterns while others exhibit none (or even an anti-correlation) across similar-sized two-dimensional chromosomal regions [73]. Initial analysis found that, within a co-regulated gene family, such as the RRB regulon members, the clustered members of the family were localized to genomic loci that had a higher transcriptional correlation which extended across a greater chromosomal region than the singleton (non-clustered) members of the family [74].

One interpretation from all of this data is that, during the normal course of genome evolution, whenever two family members are clustered together, there is a selective advantage exerted to maintain this positioning. This can result from multiple possible driving factors, including the minimization of toxic intermediates and more efficient resource management, but these are just two of many possibilities. The prevalence, significance, and analysis of the functional clustering of metabolically related genes across the *Ascomycete* lineages are the subject of a number of reviews on this topic [35,75]. Throughout the rest of this manuscript, we will focus on the current state of analysis and characterization surrounding the functional clustering of metabolically related genes as a conserved genomic organizational feature across the *Basidiomycetes*.

## 3. *Basidiomycetes* Are the Understudied Member of the *Dikarya* Clade

*Basidiomycetes* represent a distinct phylum within the *Dikarya* sub-kingdom, and membership within this clade includes species that are pathogens, symbionts, and decomposers [76]. *Basidiomycetes* represent between 32–34% of all described fungi, with this phylum second only to the *Ascomycetes* for the number of scientifically characterized species [1,77,78]. Estimates suggest that approximately 40,000 unique species have been described thus far, with the potential of there being up to 4.2 million *Basidiomycete* species globally, indicating that there is a wealth of diversity yet to be described and characterized [79].

The life cycle of *Basidiomycetes* can vary considerably and represents evolution and adaptation to the specific environmental and pathogenic niche occupied. Many members of this phylum have a dimorphic life cycle, with many of the unicellular species able to exist in alternating forms, including as a monokaryotic yeast form that can undergo budding or fission to divide and transition into a dikaryotic filamentous form characterized by the growth of long, branching hyphae [80]. This ability to transition between lifestyles is linked to pathogenicity [80]. Similar to other fungi, members of this phylum can reproduce sexually through spores, called basidiospores, stored in a specialized structure called the basidia, from which this clade derives its name.

Due to the varied life cycles of *Basidiomycetes*, they produce an incredibly diverse repertoire of metabolic compounds with myriad pharmaceutical and biotechnological applications. Decomposers and agricultural pathogens produce enzymes that are incredibly efficient at degrading cell wall materials in plants [81,82]. Two of these enzyme families are those that degrade polysaccharides via hydrolysis (e.g., xylanases and cellulases) and those that can degrade lignin and open phenol rings (e.g., laccases, ligninases, and peroxidases) [83]. This makes fungi in this clade incredibly important members that contribute to the environmental carbon cycle [84]. *Basidiomycetes* also produce a variety of second metabolites and natural products with many diverse bioactive properties. Some representative bioactive molecules include: sesquiterpenoids, polyketides, vibralactones, triterpenoids, sterols, carboxylic acids, and saccharides [85]. There is exceptional potential for fungal-derived molecules to be used in the treatment of diseases and thus enhance health [86].

## 4. The Incidence and Prevalence of Functionally Related Gene Clusters across the *Basidiomycetes* Lineages

The *Basidiomycetes* are subdivided into four subphyla: *Agaricomycotina*, *Pucciniomycotina*, *Ustilaginomycotina*, and *Wallemiomycotina* [87]. Within and between these subphyla, incredible diversity can be found among the described species. Some members have been studied for long periods of time, such as the corn smut *Ustilago maydis* and the opportunistic human pathogen *Cryptococcus neoformans*, and there is a wealth of data available. Other members are much less well-characterized, although, with the wealth of bioactive molecules of broad value that are abundant across these clades, there have been numerous advances and opportunities to gain a better understanding of them. There are many secondary metabolites described and identified, and many more predicted as a source of novel, yet-to-be-characterized compounds with therapeutic and pharmaceutical potential [88].

### 4.1. Ustilaginomycotina

The majority of this subphylum that has been described thus far represents dimorphic plant parasites, alternating between a yeast form that is haploid and a parasitic dikaryotic phase [89]. This subphylum contains one of the best-studied and characterized *Basidiomycetes*, *Ustilago maydis* (Supplementary Figure S3). *U. maydis* is commonly used as a pathogenic model for hemibasidiomycete fungi, of which there are more than 1500 species, including many economically important plant pathogens [90]. *Ustilago maydis*, commonly known as corn smut, is a fungal pathogen that induces the formation of tumors in maize (an important plant crop) [91]. This fungus is only infectious to maize and the closely related teosinte and is a biotrophic pathogen, keeping the host cell alive during successful colonization [92,93].

This species forms characteristic teliospore, which is a spherical structure seven micrometers in diameter, covered with rounded cones that protrude from the surface (Supplementary Figure S3). This species only becomes infectious upon the fusion of two hyphae, which spontaneously grow from the teliospores (Supplementary Figure S3). Upon mating, two cells produce a dikaryon that develops into infectious hyphae. *U. maydis* will then form specialized infection structures, called appressoria, to invade the cell walls and membranes of the plant. Upon infection, the fungus will form many black spores, called “smut”, from which the common name is derived.

*U. maydis* had its entire genome sequenced and it was determined that the genome is predicted to contain some 6902 protein-coding genes distributed throughout its 23 chromosomes. Due to its life cycle, *U. maydis* has evolved a unique secretome that minimizes damage to the host cell. This prevents fragmentation of the cell wall, which can induce a robust plant defense response [94]. Interestingly, of the 426 predicted proteins that comprise this secretome, approximately 20% are found to be organized into 12 functionally related gene clusters spread throughout the genome [95].

**Figure 2:**
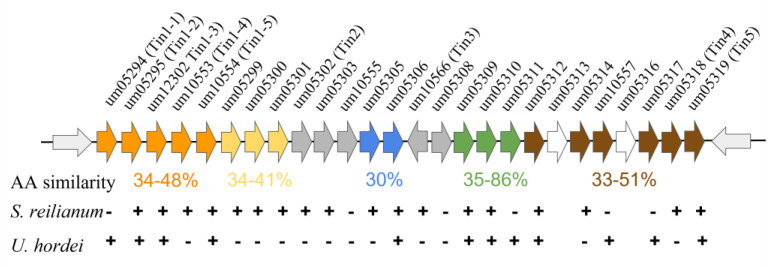
The *Ustilago maydis* gene ‘supercluster’. The supercluster of secretory protein-coding genes as depicted at their endogenous chromosomal locus, chromosome 19. The distinct groupings are color-coded based on whether they exhibit similar amino acid sequences within the supercluster itself, and the amino acid similarity is presented below each cluster. Conservation of the *U. maydis* genes within the related smut fungi *S. reilianum* and *U. hordei* is indicated below each gene indicating conservation (+) or lack of conservation (−). Not all syntenic relationships are maintained within each organism. Figure and data adapted from [96].

The initial characterization of these clusters revealed that they vary in size from 2–12 genes and that the genetic manipulation of these clusters alters the infectious ability of this smut [95]. Subsequent analysis has shown that these clusters vary in size up to 24 protein-coding genes, which differ in expression based on the site of fungal infection in the host, and that these genes can profoundly alter infectivity, including tumor formation [96]. Chromosome 19 contains a ‘supercluster’ consisting of 19 genes that code for secretory proteins, many of which are required for the successful infection of the host (Figure 2). This region is interesting as these genes can be subdivided into five distinct groupings based on homology, and many are conserved across evolutionarily divergent fungi [96]. This shared homology may indicate a shared past that gave rise to multiple clusters at the locus due to gene duplication or recombination events. Once these clusters have formed, they remain linked, potentially to modulate expression these distinct gene families simultaneously.

The systematic genetic dissection of the secretory genes found throughout the genomes as clusters revealed their importance to virulence in *U. maydis*. After analysis of the loss of function deletions throughout the *U. maydis* secretory protein gene clusters, it was observed that the disease-associated phenotypes ran the gamut from increased virulence to decreased virulence and altered tumor formation and growth upon infection of the host, indicating that some genes within these pathogenic clusters limit the severity of the infection to aid host survival.

Transcriptional profiling during infection has identified coordinately expressed transcripts that are linked to pathogenicity, such as the *MIG1* and *MIG2* gene clusters. These genes share similarities with *AVR* genes, the avirulent gene family from *Cladosporium fulvum* (an *Ascomycete*) [90]. The *MIG2* gene codes for a secretory protein and is a component of a transcriptionally co-regulated six-gene cluster. These genes are not expressed during fungal growth in the yeast form; however, induction occurs upon the penetration of the pathogen into the host cell [97]. Functional clustering of pathogenicity-regulated transcripts is a feature of *U. maydis*. This may facilitate balancing the necessary response upon infection to allow for the successful colonization of the host, maximizing the likelihood of both the host and the pathogen surviving and allowing for the tighter production of molecules with potentially damaging effects on survival.

In addition to serving as a model for fungal infections, *U. maydis* produces mannosylerythritol lipids (MELs)—secondary metabolites that are of interest due to their antimicrobial, cosmetic, and anti-aging properties [98]. They are excellent surfactants and have applications in the potential treatment of leukemia, schizophrenia, and the metabolic dysfunction of dopamine due to their demonstrative anti-inflammatory properties [99]. One of the major sources of MELs is from production and secretion in *U. maydis*. The genes that code for mannosylerythritol lipids are induced upon nitrogen starvation and are co-localized as a five-gene cluster: *MAT1-MMF1-MAC1-EMT1-MAC2* (Supplementary Figure S4A) [100]. These genes share synteny with another cluster (a doublet) of unknown function in *U. maydis*. This suggests the possibility of gene duplication and maintenance as a cluster, potentially for regulatory purposes [100].

*U. maydis* also produces a class of molecules called siderophores that are required for iron acquisition and storage [101]. This fungus produces two different derivatives known as ferrichrome and ferrichrome A—cyclic peptides produced by non-ribosomal peptide synthases (NRPs). The production of ferrichrome in this fungus is dependent on the ornithine monooxygenase, *Sid1,* and the NRP, *Sid2* [102,103]. The *sid1* and *sid2* genes represent a divergently transcribed, non-ribosomal peptide synthetase gene cluster that are co-regulated across a 3.7 kB intergenic region that is coordinately regulated by iron (Supplementary Figure S4B) [103].

Ustilagic acid (UA) is a cellobiose lipid (CL) that exhibits antibacterial and antifungal activity [104]. There is a 58 kB region that contains 12 open reading frames that are co-regulated, representing a cluster that results in the biosynthesis of UA [105]. This cluster is under the transcriptional regulation of the *rua1* gene—a nuclear localizing zinc-finger transcription factor that is a member of the cluster itself [106]. Itaconic acid (IA) has industrial applications in the production of resins, acrylic plastics, and other agents [107]. In *U. maydis*, the biosynthesis of IA involves the coordinated expression of a metabolic pathway that includes a five-gene metabolic cluster, the *TAD1-ITP1-ADI1-MTT1-RIA1* gene locus [108]. This metabolic cluster is coordinately regulated via cis-regulatory sequences that induce expression throughout this cluster, resulting in the synthesis of IA in response to nitrogen depletion [109].

Other members of this clade are much less well-characterized, although functional clusters of metabolically related genes for secretory proteins and secondary metabolites have been identified. Fungi in the genus *Malassezia* colonizes the skin of humans and many warm-blooded animals. They are involved in the formation of dandruff, atopic eczema, and seborrheic dermatitis [110]. Within this genus, *Malassezia globosa* has had its genomes sequenced, revealing six clusters of genes that code for secreted proteins. These included proteins from five distinct gene families: lipase family (LIP), lipase family (LIP3), phospholipase C, aspartyl proteases, and acid sphingomyelinases, all of which may be involved in the pathogenic lifestyle of this species by allowing them to harvest fatty acids and lipids from the host [111]. *Sporisorium scitamineum* is a sugarcane smut that contains the eleven-gene cluster for CL, which shares the conservation of homology and synteny with *U. maydis* [112]. It is likely that the continued characterization and comparative analysis of *U. maydis* will expand the repertoire of functional clusters within *Ustilaginomycotina*.

### 4.2. Agaricomycotina

The subphyla *Agaricomycotina* contains over 36,000 described fungi, and its membership is populated with saprotrophs that are excellent at consuming decaying organic matter, such as mushrooms, jelly fungi, and basidiomycetous yeasts, such as *Cryptococcus neoformans* [113,114].

*Cryptococcus neoformans* (and many *Basidiomycete* species) contain the galactose metabolic gene cluster, *GAL10-GAL1-GAL7*. This metabolic gene cluster is extensively conserved, from the closely related *Cryptococcus grubii*, *Cryptococcus gatti*, and *Tremelia mesenterica*, through the more distantly related *U. maydis* [115,116]. Interestingly, the *GAL* gene clusters appear to have evolved throughout fungal lineages multiple times independently, utilizing different methods [115]. This includes the formation of the current *GAL* cluster seen in *Saccharomyces* and *Candida* lineages through the genome rearrangement of genes that were initially unclustered, the formation of the *GAL* cluster seen in *Schizosaccharomyces* lineage via horizontal gene transfer (from *Candida* yeasts), and an independent clustering forming within the *Cryptococcus* lineage [115]. In each case, this may ultimately be driven by the cytotoxic effects of Gal-1-P favoring the co-localization of the enzymes as a cluster to balance expression (specifically limiting the stochastic effects on gene expression), providing a selective mechanism that converges on this genomic arrangement.

*C. neoformans* has other co-regulated gene clusters for primary metabolite consumption. A non-canonical metabolic input is the amino sugar *N*-acetyl-d-glucosamine (GlcNAc). GlcNAc is capable of being utilized for energy and can be metabolized and broken down by many fungi. The catabolic pathway has been elucidated in *C. neoformans*, and the four putative genes necessary for this process have been identified: CNAG_06098 (Nag1), CNAG_06186 (Ngt1), CNAG_06190 (Dac1), and CNAG_06191 (Hxk3) [117]. These four genes are co-expressed and have been found to cluster together along the genome, with *DAC1* and *HXK3* as physically adjacent neighbors [117]. Additionally, the three genes that correspond to high-affinity iron uptake, *FET3-FTR1-URF1*, are clustered together in a triplet within *C. neoformans*, although this clustering is not observed within *S. cerevisiae* [118]. The significance of this observation is not known at present, illustrating the need for further study and analysis via many approaches, including the computational, bioinformatic, and functional dissection of the genetics of this cluster and myriad others as well.

Elsewhere in this clade, there are examples of species that have clusters of detoxification enzymes, which are essential for survival in decaying matter. The crust fungus, *Phanerochaete chrysosporium*, contains 16 gene clusters of the P450 gene family, with as many as 11 members clustered at a single locus [119]. Consistent with this organism’s consumption of decaying wood, this species also has a trio of clustered genes that comprise the cellobiohydrolase gene cluster, which degrades cellulose by hydrolyzing 1,4-β-D-glycosidic bonds [120]. Within the multicopper oxidase gene family, *MCO1*, *MCO2*, and *MCO3* are co-localized as a trio, and a fourth member, *MCO4*, is found about 11 kB away from this triplet [121]. Additionally, the lignin peroxidase gene family has six members that are clustered as pairs throughout the genome: *LIPA-LIPB*, *LIPI-LIPG*, and *LIPH-LIPJ* [122]. The lignin peroxidase gene cluster is composed of three highly homologous genes, named *CRO3*, *CRO4*, and *CRO5* upon their characterization [123].

The more distantly related *Tapinella panuoides* is a poisonous, wood-degrading mushroom within this clade. *T. panuoides* produces two enzymes that collaborate in the synthesis of the terphenylquinone atromentin from L-tyrosine, which is central to the formation of pigments across various clades [124]. Atromentin has several potential applications, including its use as an anticoagulant and smooth muscle stimulant [125,126]. The biosynthetic enzymes *ATRD* and *ATRA* are found as a gene cluster separated by a putative alcohol dehydrogenase coding gene [124]. Taken together, it is clear that secondary metabolite gene clusters and metabolic gene clusters are conserved throughout this clade; however, there are significant areas that are ripe for exploration and functional dissection.

This clade includes several species of fungi that are known to be edible and medicinal. *Ganoderma lucidum* and *Ganoderma applanatum* are two members of this clade that have been widely reported for their medical uses and are a source of numerous bioactive compounds [127]. These species are colloquially known as white rot fungus, which have been utilized for medical purposes for thousands of years in China, Japan, Korea, and other Asian countries [128]. Known by the common name Reishi, these organisms have been used in traditional Chinese medicine for the treatment of numerous maladies, including anti-atherosclerotic, anti-inflammatory, analgesic, antioxidative, anti-aging, and anti-cancer effects. It is estimated that there are over 400 distinctive bioactive compounds within these species. Several classes of these compounds demonstrate anti-tumor properties, including the following: beta-D-glucans, cerebrosides, nucleotides, sterols, steroids, and triterpenes [128,129]. With the whole genome of this organism having been sequenced, initial characterization identified 24 distinct clusters of the cytochrome P450 monooxygenase gene family scattered throughout the 12 chromosomes [130]. There is likely to be numerous other clustered biosynthetic gene clusters that will be identified during follow-up analysis and study.

*Auricularia auricula-judae* is nutrient-rich, a source of numerous carbohydrates, and produces numerous antioxidants. Medical uses of this mushroom include promoting wound healing (as it has been shown to increase fibroblast and keratinocyte proliferation) and increasing collagen synthesis [131]. *Lentinula edodes*, better known by the common name Shiitake, is globally quite popular for its taste and nutrition. *L. edodes* produces polyacetylenes and sulfur compounds, many of which display antimicrobial activity [132]. *Flammulina velutipes*, commonly referred to as enokitake and widely consumed nutritionally, is a source of bioactive terpenes [132]. High-quality reference genomes for *L. edodes* and for *F. velutipes* have both been obtained; however, initial analyses did not focus on the genomic distribution and clustering of metabolically related co-expressed genes [133,134]. These resources are ripe for further analysis and functional dissection on a genetic and genomic level. While systematic analyses have yet to be performed, more and more tools are becoming available, including genome sequences for *Trametes villosa* (a source of lignin-degrading enzymes such as laccase and manganese peroxidase)*, Tremella yokohamensis*, and *Tremella fuciformis* [135,136]. The availability of these resources, combined with further sequencing efforts, will allow for extensive comparative genomic analysis.

### 4.3. Pucciniomycotina

The subphyla *Pucciniomycotina* is composed of many plant, animal, and fungal pathogens. There are over 8000 described species; however, the overwhelming majority (90%) represent plant pathogens commonly called rust fungi [137]. There are nine classes and twenty orders that have been described within this taxa [138,139,140]. This clade is filled with species that are obligate biotrophs unable to survive outside of their hosts, and they have extensive adaptations on a genomic level related to this lifestyle [141]. These adaptations include expanded gene families for secreted proteins and transport proteins predicted to play an integral role in pathogenicity and infection [141].

*Microbotryum lychnidis-dioicae* is an anther smut that infects and sterilizes flowers, producing spores that are spread by pollinators that visit the plant [142]. This fungus serves as a model for infectious disease, host shifts, and the characterization of pathogenicity and the associated genes [143]. The genome of *M. lychnidis-dioicae* contains several genes that are the result of tandem gene duplications, including several secretory protein families which appear to have been expanded by gene duplication. The MVLG_04105 family consists of four members, three of which are found clustered on the mating chromosome [144]. Two glyoxal oxidase domain-containing genes, which catalyze the oxidation of aldehydes to carboxylic acid, are found clustered together [144].

*Mixia osmundae* is a rare pathogen that has been isolated on ferns that are native to Japan, the United States, China, and Taiwan [145,146]. This fungi is an intracellular parasite of *Osmunda* and *Osmundastrum* ferns [146]. This species infects the host cells, causing the plant to form brown-yellow lesions and develop spores, which appear as a powdery layer upon development [147]. There is a high-quality reference genome for this species, *M. osmundae*, which exhibits a compact genome estimated to be around 13.6 mega-bases in size, contains few repetitive regions, and has a high gene density [140]. There is much to be learned as many of the known fungal virulence genes are not conserved (3/51 queried), several enzyme families have uncharacterized functions, and there are many P450 cytochromes. This resource is ripe for dissection and analysis, and as efforts to sequence diverse fungal lineages expand, there will be ample opportunities for analysis to further our understanding of the prevalence and significance of the functional clustering of co-regulated genes within this subphyla [140].

### 4.4. Wallemiomycotina

One of the most enigmatic subphyla within this clade is that of the *Wallemiomycotina*. Representative members are chiefly characterized by the xerophiles subspecies (from Greek: ‘dry loving’). Xerophiles have a great xerotolerance—the ability to survive and reproduce in environments with very low water availability—and some species grow optimally at up to 15% NaCl [148,149].

One of the better-characterized and medically relevant members of this grouping is *Wallemia mellicola*. *W. mellicola* is found worldwide thanks to its ability to grow in habitats that contain soil, dust, and plants, including food crops and vegetables such as peas, maize, and beans [149,150]. The xerotolerance of this species means that it can survive in dried foods, salty foods, and sugared foods, surviving these processes and spreading disease and contamination. Furthermore, these fungi are human pathogens and can cause diseases such as farmer’s lung disease and cutaneous and subcutaneous infections [151,152]. Farmer’s lung disease varies in severity; however, treatment only exists for the less severe, acute form of the disease, and chronic exposure presents a long-term challenge that greatly diminishes the health span of affected individuals [153]. Recently, this species had its genome sequenced, though a systematic analysis and annotation of the relationships of metabolically related, functional clusters has yet to be performed [154]. The sequence of the related species *Wallemia sebi* has been sequenced, revealing adaptations that were made to respond to osmotic stressors. Within this organism, there is a less frequent occurrence of gene duplications seen within this genome; however, one such duplication is a five-gene tandem cluster whose members code for transport proteins, the expression of which responds to osmotic stimulation [155]. While this clade is much less well-studied and characterized than the other sub-phyla, functional clusters have been identified, and further analysis will likely yield more insights into this phenomenon.

## 5. Conclusions and Perspectives

The *-omics* era has brought a newfound understanding of the tenets that underlie genome organization and transcriptional control. The functional clustering of related genes has long been recognized as a feature of prokaryotic organisms, where genomes are organized into operons. The organization of metabolically related genes in a tandem arrangement, allowing for polycistronic transcription under the regulation of common cis-regulatory sequences, is widespread and common, oftentimes taught as models of gene expression circuitry [156,157]. This organization is not a feature of eukaryotic genomes, although the clustering of secondary metabolite genes into clusters has been described [58]. True operon-like genomic structures seem to be the exception rather than the norm across eukaryotes [158,159].

The emerging picture is that there is extensive clustering of functionally related gene families throughout the genome. This lends itself to a rather straightforward model as follows: the clustering of genes allows for the stabilization of expression patterns, most likely due to position effects that are specific to individual loci. This phenomenon is prevalent throughout *Ascomycetes* and appears to be conserved throughout their *Dikaryon* brethren, the *Basidiomycetes* [35]. The *Basidiomycetes* are clearly the least studied of the two members of this subkingdom, and there is a significant need (and opportunity) for further research and study. As the number of *Basidiomycetes* within a sequenced genome increase, detailed analyses will illuminate insights throughout this clade. The number of genomes sequenced within this phylum has expanded significantly (from roughly 200 genomes in 2016 to 635 currently sequenced), leading to new opportunities for study and understanding [160,161].

**Figure 3:**
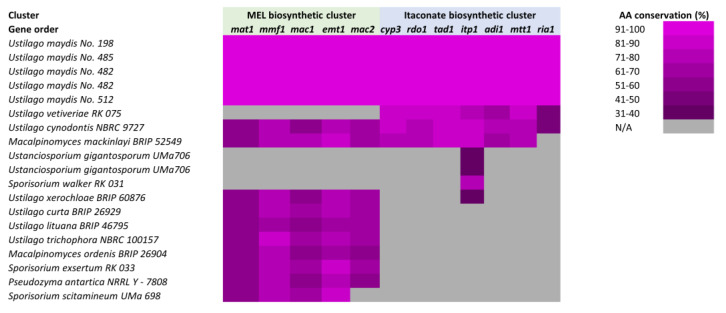
Conservation of the mannosylerythritol lipids (MEL) and itaconate biosynthetic gene clusters in *Basidiomycetes*. The conservation of the *U. maydis* MEL and itaconate gene clusters throughout related species and isolates is depicted. Heat map corresponds to the percentage of amino acid conservation for each protein within each organism, when data is available for such analysis. Figure adapted from [162].

The identification of biosynthetic gene clusters, along with their medical, pharmaceutical, and industrial applications has led to the initial characterization of functional gene clustering within less well-studied fungal lineages. New tools will enable analysis within *Basidiomycetes* and across divergent fungi to explore conservation and syntenic relationships, offering exciting opportunities for the identification of clusters in less well-studied species and providing insight into their formation and evolution. Initial efforts provide a picture of conservation in *Basidiomycetes* that is consistent with observations in *Ascomycetes.* Using the mannosylerythritol lipids (MEL) and itaconate biosynthetic gene clusters as a model, the conservation of the *U. maydis* clusters was explored in divergent *Basidiomycete* lineages (Figure 3). There is extensive conservation of synteny and amino acid composition of these clusters in closely related organisms that begins to drop off as the evolutionary distance increases. There are also larger gaps in our understanding due to a lack of genomic data available to perform more thorough and systematic comparisons.

Resources such as the Yeast Gene Order Browser provide the tools for incredibly thorough genomic comparisons and analyses [163,164]. This tool has expanded in recent years to include members of the *Candida*, *Pichiaceae*, and *Oomycete* families [165,166,167]. Hopefully, future expansions will allow for analysis across divergent *Dikarya*, including the *Basidiomycetes* and other fungi lineages.

Novel approaches are emerging that allow for the identification of functional gene clusters through myriad mechanisms, such as the observation that detoxification or protective genes are oftentimes co-localized with enzymes that synthesize potentially cytotoxic molecules [75,168]. Global analyses of fungi estimate that a third of genes may be found in clusters of one type or another [169]. Functionally related gene clusters can arise from many, oftentimes overlapping, evolutionary mechanisms, including the following: horizontal gene transfer, vertical gene duplication, meiotic sex and recombination, non-meiotic sex, ecological selection, and natural selection [170,171]. Regardless of what mechanism forms a specific cluster, it is abundantly clear that these relationships are subsequently maintained throughout a variety of metabolic and functional families [172].

## Data Availability

No new data were created or analyzed in this study. Data sharing is not applicable to this article.

## Supplementary Materials

The following supporting information can be downloaded at: https://www.mdpi.com/article/10.3390/jof9050523/s1, Figure S1: Phylogenetic relationship between model *Ascomycetes* and the *Basidiomycetes* discussed within this work, depicted as a circular tree. Figure S2: Phylogenetic relationship between model *Ascomycetes* and the *Basidiomycetes* discussed within this work, depicted as an unrooted tree. Figure S3: Electron micrographs of *Ustilago maydis*. Figure S4: Schematic of gene clusters in *Ustilago maydis* discussed in the manuscript. Table S1: Representative members of the phylum *Basidiomycota* with annotations of research, pharmaceutical, and clinical relevance. Refs. [173,174,175,176,177,178,179,180,181] are cited in the supplementary material.
