# Supplementary material for: Functional Clustering of Metabolically Related Genes Is Conserved across Dikarya
Source: J Fungi (Basel). 2023 Apr 28;9(5):523. doi: 10.3390/jof9050523 (PMC10218983; doi:10.3390/jof9050523)
Supplement: Supplementary file 1 [file jof-09-00523-s001.zip › jof-2283655-supplementary.pdf]

Supplemental Figure S1: Phylogenetic relationship between model *Ascomycetes* and the *Basidiomycetes* discussed within this work, depicted as a circular tree. This tree was generated utilizing the Interactive Tree of Life tool, using NCBI taxonomy inputs as described in Figure 1 [1].

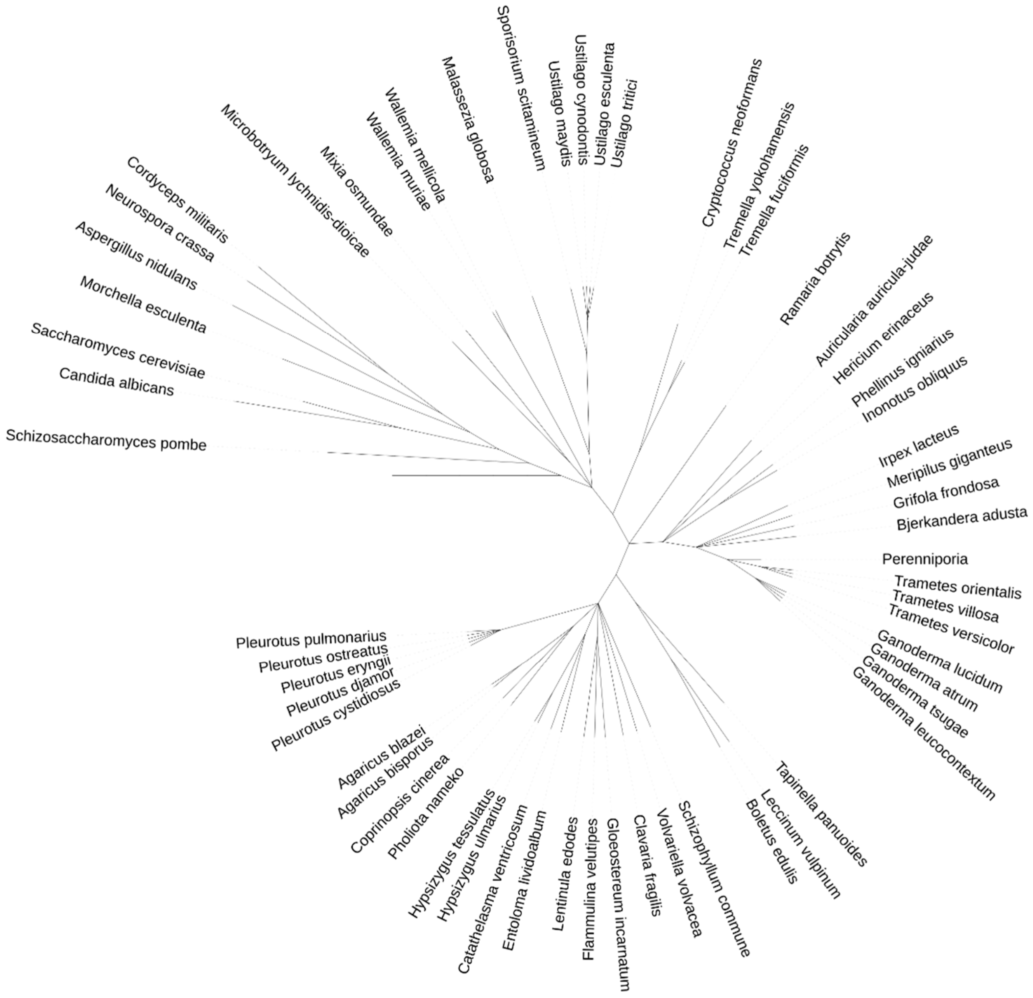

Supplemental Figure S2: Phylogenetic relationship between model *Ascomycetes* and the *Basidiomycetes* discussed within this work, depicted as an unrooted tree. This tree was generated utilizing the Interactive Tree of Life tool, using NCBI taxonomy inputs as described in Figure 1 [1].

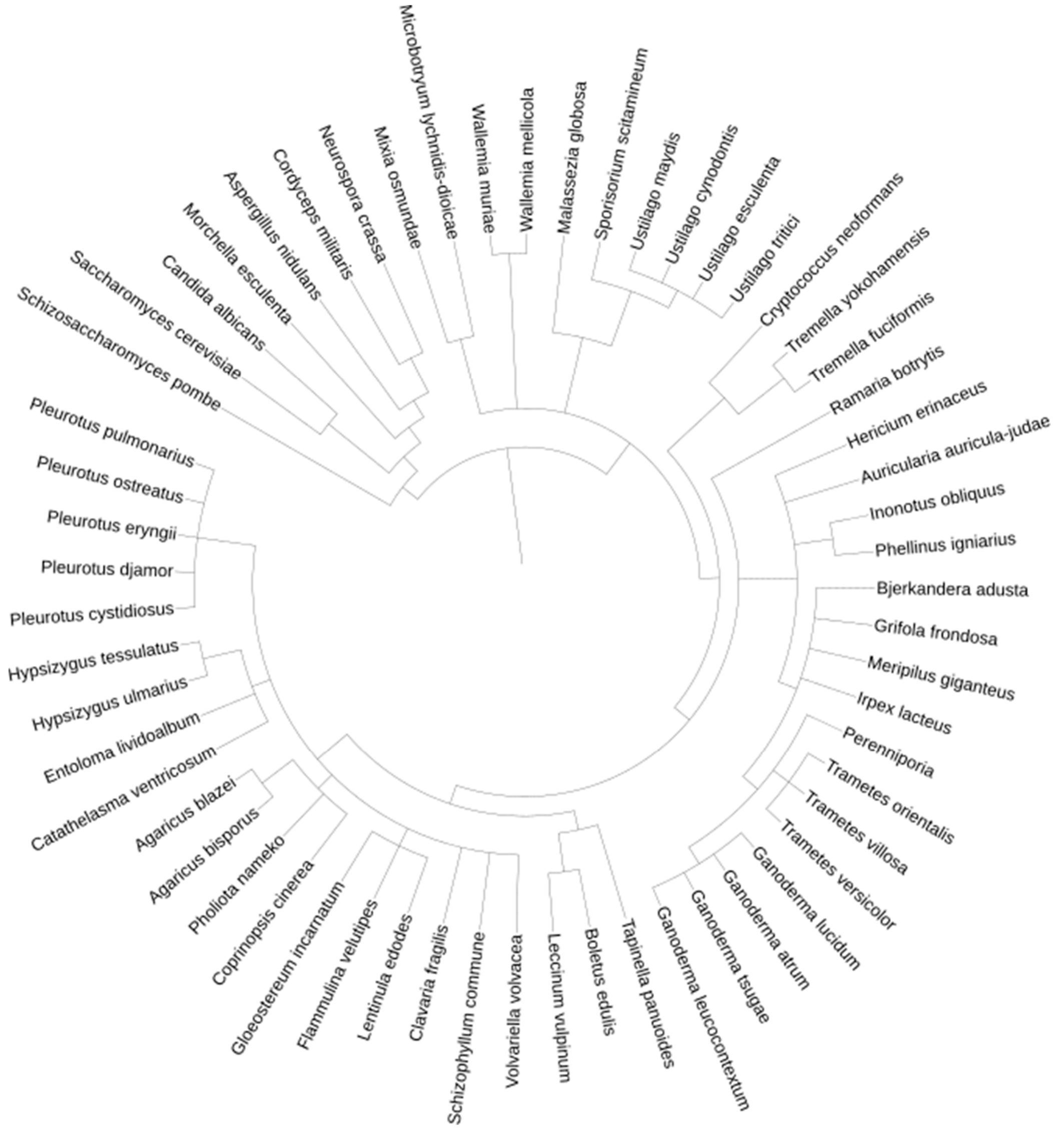

Supplemental Figure S3: Electron micrographs of *Ustilago maydis*. The teliospore of *U. maydis* depicting the characteristic spherical shape that is decorated with rounded cones protruding (top, middle). Branching hyphae spontaneously grows from the teliospore, which can fuse with another to form a dikaryon (bottom) and develop into the infectious appressorium.

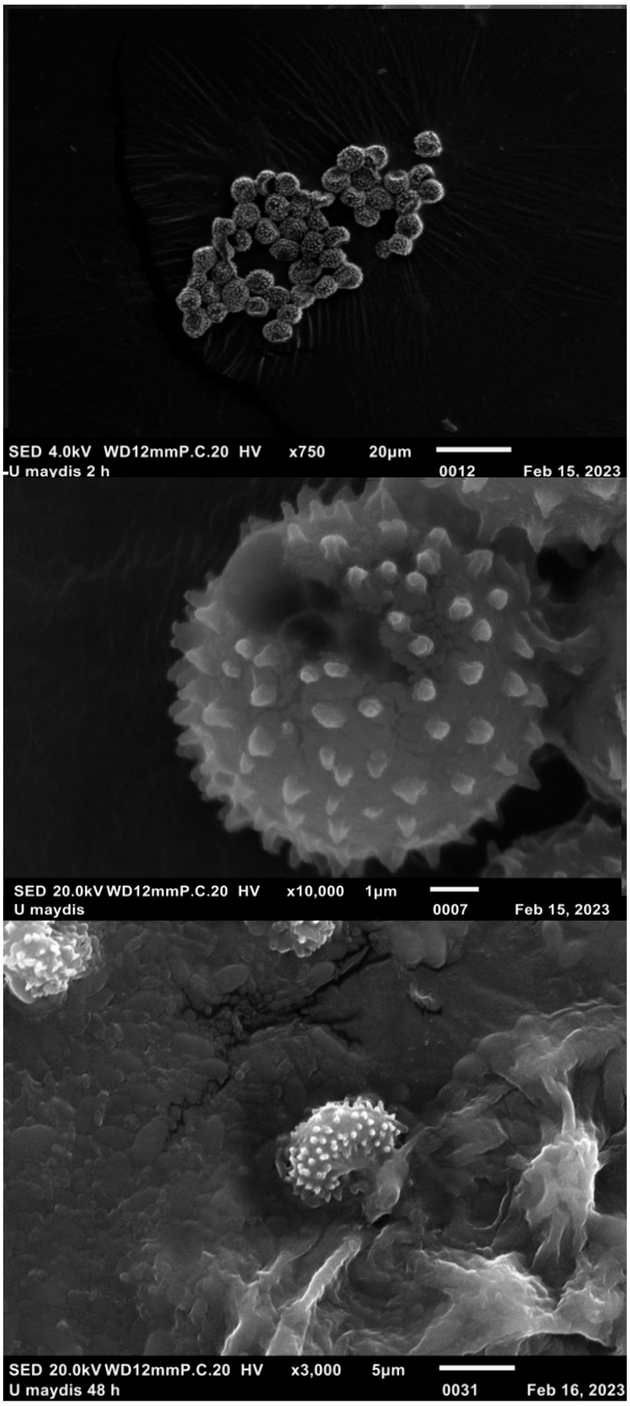

Supplemental Figure S4: Schematic of gene clusters in *Ustilago maydis* discussed in the manuscript. A. the *MEL* biosynthetic gene cluster and B. the *SID1/2* divergently transcribed gene cluster. Loci schematics are not to scale, but are used to depict the relative arrangement and genomic distribution of the genes referenced in the text. Loci schematics were as described in [2,3].

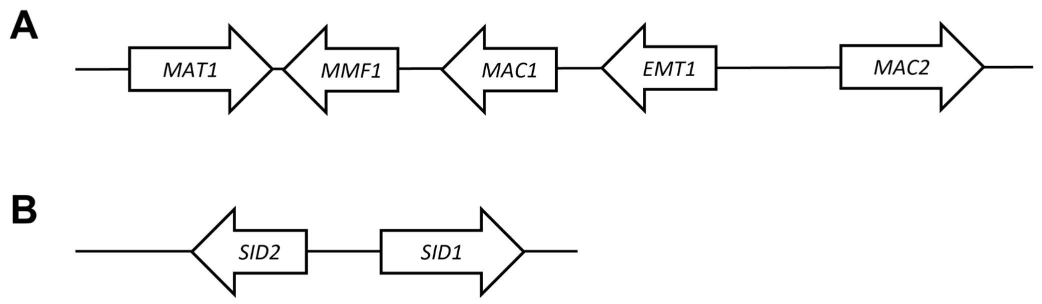

**Supplemental Table S1: Representative members of the phylum *Basidiomycota* with annotations of research, pharmaceutical, and clinical relevance**			
**Category:**	**Reference (listed below)**	**Species Name:**	**Comment:**
Model Organisms	[7]	*Ustilago maydis*	Corn smut
	[7]	*Coprinopsis cinerea*	Gray shag
	[7]	*Schizophyllum commune*	Splitgill mushroom
Associated with disease	[4]	*Bjerkandera adusta*	Chronic cough
	[4]	*Cyclomyces tabacinus*	Soft-tissue Infection
	[4]	*Ceriporia lacerata*	Fungal pneumonia
	[4]	*Hormographiella aspergillata*	Pulmonary infection and Acute myeloid leukemia
	[4]	*Inonotus tropicalis*	X-linked chronic granulomatous disease
	[4]	*Irpex lacteus*	Pulmonary abscess
	[4]	*Perenniporia*	Invasive pulmonary infection
	[4]	*Phellinus undulates*	Soft-tissue Infection in heart
	[4]	*Schizophyllum commune*	Sneezing, nasal obstruction, coughing
	[4]	*Sporotrichum pruinosum*	Pulmonary infection and pulmonary abscess
	[4]	*Volvariella volvacea*	Invasive brain disease
	[5]	*Wallemia mellicola*	Farmers Lung Disease
Pharmaceutical applications	[6]	*Agaricus bisporus*	Anticancer, Immunomodulatory, Kidney tonic
	[6]	*Agaricus subrufescens*	Anticancer
	[6]	*Ganoderma spp.*	Antiinflammatory, anticancer, antiviral, antibacterial, Blood pressure regulation, cardiotonic, Immunomodulatory, Kidney tonic, Hepatoprotective, Nerve tonic, Sexual potenting, Antiasthmatic
	[6]	*Grifola frondosa*	Antifungal, Anticancer, antiviral, antibacterial, blood pressure regulation, cholesterol lowering, antidiabetic, Immunomodulatory, Hepatoprotective
	[6]	*Hericium erinaceus*	Anticancer, Immunomodulatory, Nerve tonic, Antiasthmatic

	[6]	*Lentinula edodes*	Antiinflammatory, anticancer, antiviral, antibacterial, Blood pressure regulation, Cholesterol lowering, Antidiabetic, Immunomodulatory, Kidney tonic, Hepatoprotective, Sexual potenting
	[6]	*Pleurotus ostreatus*	Anticancer, antiviral, antibacterial, Cholesterol lowering, Immunomodulatory, Nerve tonic
	[6]	*Trametes versicolor*	Anticancer, antiviral, antibacterial, Kidney tonic, Hepatoprotective
	[6]	*Schizophyllum commune*	Antiinflammator, anticancer, antibacterial, Immunomodulatory, Kidney tonic, Hepatoprotective
	[6]	*Volvariella volvacea*	Anticancer, antiviral, antibacterial, Cholesterol lowering
Antioxidant properties and product production	[8]	*Agaricus bisporus*	Elimination of free radicals
	[8]	*Agaricus blazei*	Inhibition of enzymatic oxidative processes
	[8]	*Boletus edulis*	DPPH anti-radical, anti-hydroxyl, anti-nitric activity of the anionic anti-superoxide radical
	[8]	*Catathelasma ventricosum*	Increased GSH-Px, Superoxide dismutase and Catalase; decreased MDA formation, Increased activity of antioxidant enzymes in the liver and kidneys
	[8]	*Clavaria fragilis*	Elimination of free radicals
	[8]	*Flammulina velutipes*	Elimination of free radicals
	[8]	*Entoloma lividoalbum*	Decreased GSH, increased GSSH, reduction of MDA formation
	[8]	*Ganoderma leucocontextum*	Inhibition of free radicals induced by H2O2
	[8]	*Ganoderma lucidum*	Removal of hydroxyl radical and anion superoxide, Inhibition and elimination of DPPH radical
	[8]	*Gloeostereum incarnatum*	Increased levels of superoxide dismutase in serum and spleen, decreased levels of reactive oxygen species in the spleen
	[8]	*Hohenbuehelia serotina*	Elimination of ABTS and hydroxyl radicals
	[8]	*Hypsizygus tessulatus*	Elimination of free radicals
	[8]	*Hypsizygus ulmarius*	Elimination of free radicals
	[8]	*Inonotus obliquus*	Elimination of superoxide and hydroxyl radicals and free radicals, increased activity of antioxidant enzymes

	[8]	*Lentinula edodes*	Elimination of free radicals
	[8]	*Meripilus giganteus*	Reduce MDA formation
	[8]	*Pleurotus cystidiosus*	Decreased GSH; Increased GSSH; Decrease of lipid peroxidation
	[8]	*Pleurotus djamor*	Elimination of free radicals
	[8]	*Pleurotus eryngii*	Elimination of free radicals
	[8]	*Pleurotus ostreatus*	Elimination of free radicals, decrease of ferric iron
	[8]	*Pleurotus porrigens*	Decrease in plasmatic Fe3+, decrease of iron deposited in liver (mice)
	[8]	*Pleurotus pulmonarius*	Elimination of free radicals
	[8]	*Ramaria botrytis*	Elimination of free radicals
	[8]	*Tylopilus ballouii*	Inhibition of superoxide and hydroxyl radicals
Antiinflammatory properties and product production	[8]	*Cordyceps militaris*	Inhibition of expression of pro-inflammatory genes, inhibition of the inflammatory phase of nociceptive response induced by formalin, reduction of total leukocyte migration
	[8]	*Ganoderma lucidum*	Increased expression of IFN-γ mRNA, reduction of COX-2 expression in the lung tumor model, reduction of TGF-β production in serum, increased expression of IL-12 and IFN-γ mRNA and decreased mRNA of IL-6, IL-10, COX-2 and TGF-β
	[8]	*Hericium erinaceus*	Reduction of interleukin-1β, interleukin-6 and tumor necrosis factor levels; Suppression of reactive nitrogen species and down-regulation of inducible nitric oxide synthase (iNOS) and p38 MAPK.
	[8]	*Pleurotus sajor-caju*	Inhibition of inflammation induced by formalin, decreased number of total leukocytes and myeloperoxidase
	[8]	*Trametes orientalis*	Stimulated the phagocytic function of macrophages, promote expression of serum cytokines
	[8]	*Tylopilus ballouii*	Reduction of inflammatory effect on the edema
Antitumor properties and product production	[8]	*Antrodia camphorate*	Induction of cell death by apoptosis, increased activation of caspase-3/7, inhibition of Bcl-2 protein expression, increased expression of Bax and p53 promoters of apoptosis, inhibition of the JAK/STAT3 signaling pathway
	[8]	*Antrodia cinnamomea*	Relief of prostatic epithelial hyperplasia and collagen deposition, positive regulation of IL-1, COX-2 and CD68, negative regulation of N-cadherin and vimentin

	[8]	*Cordyceps militaris*	Induction of cell death by apoptosis, decreased lipid peroxidation
	[8]	*Ganoderma atrum*	Induction of cell death by apoptosis, increased levels of intracellular cAMP and protein kinase A activity, PSG-1 enhanced the antitumor immune response, increased macrophage phagocytosis and proliferation of lymphocytes in the spleen, increased serum concentrations of TNF-α, IFN-γ and IL-2
	[8]	*Ganoderma tsugae*	Reduction of viability of cancer cells, induction of necrosis, apoptosis or differentiation
	[8]	*Inonotus obliquus*	Cell cycle arrest at G1, decreased protein expression of CDK2, CDK4 and cyclin D1, increased expression of p21, p27, and p53 and Rb and E2F1 phosphorylation
	[8]	*Leccinum vulpinum*	Decreased cell proliferation and induction of apoptosis, increased DNA damage in cells
	[8]	*Lentinula edodes*	Induction of cell death by apoptosis, increased expression of caspase-7, increased Bax/Bcl-2 ratio
	[8]	*Morchella esculenta*	Cytotoxicity of ergosterol and octadecanoic acid and induction of apoptosis
	[8]	*Phellinus igniarius*	Cell cycle arrest in G0/G1, mitochondrial membrane potential collapse, apoptosis by activation of caspase-9, -3 and PARP cleavage, bax expression
	[8]	*Phellinus linteus*	Induction of apoptosis, attenuated expression of NF-κB, β-catenin and mitogen-activated protein kinase (MAPK) proteins
	[8]	*Pholiota nameko*	Cell cycle arrest at G1, alteration of mitochondrial membrane potential, release of cytochrome c, activation of caspase-9 and caspase-3
	[8]	*Pleurotus eryngii*	Cell cycle arrest and extensive apoptosis, begative regulation of cyclin B, cyclin E and cdc-2, positive regulation of p53 and c-PARP
	[8]	*Pleurotus ostreatus*	Induction of cell death by apoptosis
	[8]	*Trametes versicolor*	Induction of cell death by apoptosis
Misc. and uncategorized	[9]	*Cryptococcus gattii*	Cerebral cryptococcal infection
	[9]	*Cryptococcus grubii*	Cryptococcal meningitis
	[9]	*Cryptococcus neoformans*	Cryptococcal infection
	[10]	*Cryptococcus bacillisporus*	Rare cause of cryptococcosis
	[11]	*Ustilago esculenta*	Smut fungus, inducing formation of culm galls

	[12]	*Ustilago tritici*	Causes loose smut on wheat, seed-borne disease
	[13]	*Ustilago cynodontis*	pH-tolerant itaconic acid producer
	[14]	*Wallemia ichthyophaga*	Extremely halophillic
	[16]	*Ganoderma lucidum*	polysaccharides, triterpenoids, proteins, peptides, adenosine and nucleosides
	[17]	*Auricularia auricula-judae*	antioxidant activity, antitumor activities, wound healing
	[18]	*Flammulina velutipes*	bioactive terpenes
	[18]	*Lentinula edodes*	antimicrobial activity
	[15]	*Wallemia muriae*	Halotolerant and allergological conditions (including bronchial asthma)
**Notes:**			
Several species fall under multiple categories and have been listed multiple times to reflect this			
Anticancer' designation: evidence that the species produces a compound that demonstrates cytotoxicity in cancer tumor cells			

References

1. Ivica Letunic, Peer Bork, Interactive Tree Of Life (iTOL) v5: an online tool for phylogenetic tree display and annotation, Nucleic Acids Research, Volume 49, Issue W1, 2 July 2021, Pages W293–W296, https://doi.org/10.1093/nar/gkab301

2. Hewald, Sandra, et al. "Identification of a gene cluster for biosynthesis of mannosylerythritol lipids in the basidiomycetous fungus Ustilago maydis." Applied and environmental microbiology 72.8 (2006): 5469-5477.

3. Yuan, Walter M., et al. "Characterization of the Ustilago maydis sid2 gene, encoding a multidomain peptide synthetase in the ferrichrome biosynthetic gene cluster." Journal of Bacteriology 183.13 (2001): 4040-4051.

4. Chowdhary, Anuradha, et al. “Recognizing Filamentous Basidiomycetes as Agents of Human Disease: A Review.” Medical Mycology, vol. 52, no. 8, Nov. 2014, pp. 782–97. DOI.org (Crossref), https://doi.org/10.1093/mmy/myu047.

5. Skalski, Joseph H., et al. “Expansion of Commensal Fungus Wallemia Mellicola in the Gastrointestinal Mycobiota Enhances the Severity of Allergic Airway Disease in Mice.” PLOS Pathogens, edited by Tobias M. Hohl, vol. 14, no. 9, Sept. 2018, p. e1007260. DOI.org (Crossref), https://doi.org/10.1371/journal.ppat.1007260.

6. Sandargo, Birthe, et al. “Biological and Chemical Diversity Go Hand in Hand: Basidiomycota as Source of New Pharmaceuticals and Agrochemicals.” Biotechnology Advances, vol. 37, no. 6, Nov. 2019, p. 107344. DOI.org (Crossref), https://doi.org/10.1016/j.biotechadv.2019.01.011.

7. Taylor, J. W., et al. “Fungal Model Organisms: Phylogenetics of Saccharomyces, Aspergillus, and Neurospora.” Systematic Biology, vol. 42, no. 4, Dec. 1993, pp. 440–57. DOI.org (Crossref), https://doi.org/10.1093/sysbio/42.4.440.

8. Vieira Gomes, Débora Cássia, et al. “Antioxidant, Anti-Inflammatory and Cytotoxic/Antitumoral Bioactives from the Phylum Basidiomycota and Their Possible Mechanisms of Action.” Biomedicine & Pharmacotherapy, vol. 112, Apr. 2019, p. 108643. DOI.org (Crossref), https://doi.org/10.1016/j.biopha.2019.108643.

9. Van Der Torre, Mireille H., et al. “Systematic Review on Cryptococcus Neoformans/Cryptococcus Gattii Species Complex Infections with Recommendations for Practice in Health and Care Settings.” Clinical Infection in Practice, vol. 15, July 2022, p. 100154. DOI.org (Crossref), https://doi.org/10.1016/j.clinpr.2022.100154.

10. Silva, Carolina, et al. “Cryptococcus Bacillisporus Causing Cryptococcoma of the Beak of an African Grey Parrot (Psittacus Erithacus), Portugal.” Medical Mycology Case Reports, vol. 34, Dec. 2021, pp. 8–12. DOI.org (Crossref), https://doi.org/10.1016/j.mmcr.2021.08.006.

11. Wang, Zheng-Hong, et al. “Gene Expression in the Smut Fungus Ustilago Esculenta Governs Swollen Gall Metamorphosis in Zizania Latifolia.” Microbial Pathogenesis, vol. 143, June 2020, p. 104107. DOI.org (Crossref), https://doi.org/10.1016/j.micpath.2020.104107.

12. Quijano, Carolina Diaz, et al. “KP4 to Control Ustilago Tritici in Wheat: Enhanced Greenhouse Resistance to Loose Smut and Changes in Transcript Abundance of Pathogen Related Genes in Infected KP4 Plants.” Biotechnology Reports, vol. 11, Sept. 2016, pp. 90–98. DOI.org (Crossref), https://doi.org/10.1016/j.btre.2016.08.002.

13. Hosseinpour Tehrani, Hamed, et al. “Engineering the Morphology and Metabolism of PH Tolerant Ustilago Cynodontis for Efficient Itaconic Acid Production.” Metabolic Engineering, vol. 54, July 2019, pp. 293–300. DOI.org (Crossref), https://doi.org/10.1016/j.ymben.2019.05.004.

14. Kralj Kunčič, Marjetka, et al. “Morphological Responses to High Sugar Concentrations Differ from Adaptation to High Salt Concentrations in the Xerophilic Fungi Wallemia Spp.” Fungal Biology, vol. 117, no. 7–8, July 2013, pp. 466–78. DOI.org (Crossref), https://doi.org/10.1016/j.funbio.2013.04.003.

15. Zajc, Janja, and Nina Gunde-Cimerman. “The Genus Wallemia—From Contamination of Food to Health Threat.” Microorganisms, vol. 6, no. 2, May 2018, p. 46. DOI.org (Crossref), https://doi.org/10.3390/microorganisms6020046.

16. Sanodiya BS, Thakur GS, Baghel RK, Prasad GB, Bisen PS (2009) Ganoderma lucidum: a potent pharmacological macrofungus. Curr Pharm Biotechnol 10: 717–742.

17. Mapoung, Sariya, et al. "Skin wound-healing potential of polysaccharides from medicinal mushroom Auricularia auricula-judae (Bull.)." Journal of Fungi 7.4 (2021): 247.

18. Fukushima-Sakuno, Emi. "Bioactive small secondary metabolites from the mushrooms Lentinula edodes and Flammulina velutipes." The Journal of antibiotics 73.10 (2020): 687-696.
